# Racial and Socioeconomic Determinants of Cardiovascular Health: A Comprehensive Review

**DOI:** 10.7759/cureus.59497

**Published:** 2024-05-02

**Authors:** Pawel Borkowski, Natalia Borkowska, Shaunak Mangeshkar, Bisrat H Adal, Nikita Singh

**Affiliations:** 1 Internal Medicine, Albert Einstein College of Medicine, Jacobi Medical Center, New York, USA; 2 Pediatrics, SPZOZ (Samodzielny Publiczny Zakład Opieki Zdrowotnej) Krotoszyn, Krotoszyn, POL

**Keywords:** poor health outcomes, health-care equity, cardiovascular disease, socioeconomic factors, racial disparity

## Abstract

Cardiovascular diseases (CVDs) are the leading cause of death globally. Their prevalence and mortality rates continue to rise. This narrative review explores well-known risk factors for CVDs such as dyslipidemia, hypertension, diabetes, obesity, and smoking, and their prevalence among different racial and ethnic groups. In addition, we expand the discussion to include the impact of socioeconomic status (SES) on cardiovascular outcomes. The data demonstrate that non-Hispanic Black and Hispanic populations not only exhibit higher rates of hypertension, obesity, diabetes, and smoking but also face systemic barriers linked to lower SES, which worsen their cardiovascular outcomes. These barriers include a lack of education, lower income, higher rates of unemployment, and poor living conditions. Beyond these commonly studied factors, these groups also suffer from higher levels of food and housing insecurity and a lack of adequate insurance coverage, all of which contribute to poorer health. Additionally, there is a higher prevalence of mental health disorders, such as depression and anxiety, among these populations. This further compounds the risks and adverse outcomes associated with CVDs. It is essential to conduct further research into how SES and race influence cardiovascular health and to refine risk assessment methods. Concentrating on these aspects would make it possible to create interventions designed to meet the needs of diverse communities and strategies that could potentially reduce morbidity and mortality from CVD across populations. Moreover, this review advocates for integrating comprehensive socioeconomic data into cardiovascular health strategies, which is crucial for developing effective public health initiatives.

## Introduction and background

Cardiovascular diseases (CVDs), including coronary artery disease (CAD), cerebrovascular disease, heart failure (HF), peripheral arterial disease (PAD), and other cardiac and vascular conditions, are the primary cause of death worldwide and significantly diminish the quality of life [1, 2]. The Global Burden of Disease (GBD) 2019 Study revealed that CVD cases almost doubled worldwide, rising from 271 million in 1990 to 523 million in 2019 [3]. Additionally, deaths attributable to CVD increased from 12.1 million in 1990 to 18.6 million in 2019. Given these trends, it is probable that the burden of CVDs will continue to grow, resulting in even higher prevalence and mortality rates in the coming years. It is commonly recognized that the primary risk factors for developing CVDs include age, gender, dyslipidemia, hypertension, diabetes, obesity, and smoking [4]. Despite this, notable disparities in CVD incidence, prevalence, treatment, and outcomes persist globally. Comprehensive data underscore the influence of socioeconomic status (SES) and ethnicity on CVD outcomes, indicating significant disparities [5-7]. Addressing these disparities presents an opportunity to improve patient outcomes. Furthermore, recognizing the cultural differences in healthcare is critical for enhancing outcomes in CVD management [8]. Culturally tailored interventions are essential for increasing patient engagement and adherence to treatment regimens. This review’s objective is to examine racial and ethnic disparities in CVD outcomes in the context of socioeconomic factors.

## Review

Racial and ethnic disparities in cardiovascular risk factors

Current research underscores significant disparities in CVD risk factors across various racial and ethnic groups. The data discussed in this paragraph is summarized in Table 1. A study by Lopez-Neyman et al., involving 8,370 participants, demonstrated that hypercholesterolemia affected nearly all races and ethnic groups similarly, with prevalence rates ranging from 24.5% to 28.0% [9]. However, the authors reported notable racial disparities in other CVD risk factors. Specifically, non-Hispanic Black individuals exhibited the highest rates of hypertension at 59.3%, exceeding those of other races. Obesity and diabetes also showed notable racial variations; namely, non-Hispanic Black individuals (50.2%) and Mexican American populations (50%) reported the highest obesity rates. Diabetes prevalence was highest among Mexican American individuals (28.6%) and non-Hispanic Black individuals (26.8%). Smoking rates were markedly higher among non-Hispanic Black individuals (23.8%) than in other groups. The CDC published similar findings in 2019, demonstrating that high total cholesterol affected all races equally, with prevalence rates ranging from 10.2% to 12.6% [10]. However, disparities emerged with other CVD risk factors. Non-Hispanic Black adults exhibited the highest prevalence of hypertension at 42.1%, and both non-Hispanic Black and Hispanic populations faced disproportionately high obesity rates, approximately 47.5% and 46.9%, respectively. Diabetes followed a similar pattern, with higher rates among Hispanic individuals (21.5%) and Black individuals (19.6%). Supporting the above findings, Meadows et al. also reported significant racial discrepancies among races when analyzing CVD risk factors [11]. The authors analyzed 45,191 individuals from the Reduction of Atherothrombosis for Continued Health (REACH) Registry and found that Asian individuals had the highest rate of hyperlipidemia at 63%, and non-Hispanic Black individuals had the most significant prevalence of hypertension at 93.0%. In terms of BMI, diabetes, and smoking, Black individuals had the highest average BMI at 28.9±6.4 kg/m², rates of diabetes at 55%, and smoking rate at 20.3%. In addition, within these broader racial categories, disparities and socioeconomic differences are further pronounced in specific subgroups, such as HIV cohorts experiencing heart failure, indicating that some subgroups are disproportionately affected even within the same racial group [12]. These findings highlight the urgent need for targeted public health strategies that address CVD risk factors specific to diverse racial and ethnic groups, aiming to reduce disparities and improve health outcomes.

**Table 1:** Racial and Ethnic Disparities in Cardiovascular Risk Factors ^a^Variable not included in the cited study. ^b^The study included patients with established atherothrombotic disease. ^c^Reported in the study as mean BMI ± SD, not percentage.

Study	Race	Racial and Ethnic Disparities in Cardiovascular Risk Factors				
		Hyperlipidemia, %	Hypertension, %	Obesity, %	Diabetes, %	Smoking, % (currently)
Lopez-Neyman et al. [9]	Black	23.8	59.3	50.2	26.8	23.8
	Hispanic	24.7	43.6	45.5	25.4	13.5
	White	27.4	43.6	38.9	14.6	18.9
	Asian	28	42.7	12.1	23.1	9.2
Centers for Disease Control and Prevention [10]	Black	10.2	42.1	47.5	19.6	N/A^a^
	Hispanic	11.2	29.4	46.9	21.5	N/A^a^
	White	12.6	28.7	38.2	13	N/A^a^
	Asian	10.7	27.2	12.4	14.5	N/A^a^
Meadows et al.^b^ [11]	Black	51.4	93	N/A^c^	55	20.3
	Hispanic	56.6	80.3	N/A^c^	53.3	13.5
	White	60.1	79.1	N/A^c^	38.8	18.1
	Asian	63	83.8	N/A^c^	50.8	13.4

Racial and ethnic disparities in cardiovascular outcomes

CVDs are the leading cause of death in the United States [13, 14]. They remain the predominant cause of mortality among African American, American Indian, Alaska Native, Hispanic, and White men [15]. For women, particularly those from Pacific Islander, Asian American, American Indian, Alaska Native, and Hispanic backgrounds, heart disease is the second leading cause of death after cancer [15]. In response to these significant health disparities, the American Heart Association (AHA) has established a new mission and a 2030 Impact Goal to increase healthy life expectancy for everyone, regardless of racial and ethnic background, economic status, or other demographic and geographic factors [16].

Kyalwazi et al. conducted a comprehensive analysis using data from the Centers for Disease Control and Prevention’s Wide-Ranging Online Data for Epidemiologic Research (CDC WONDER) database, which encompassed 17,357,312 subjects, to examine trends in CVD mortality between 1999 and 2019 [17]. Their findings revealed a significant decline in age-adjusted CVD mortality rates among both Black and White populations in the United States. Specifically, mortality rates decreased from 602.1 to 351.8 per 100,000 for Black women and from 447.0 to 267.5 per 100,000 for White women. Similarly, for men, the rates dropped from 824.1 to 526.3 per 100,000 for Black individuals and from 637.5 to 396.0 per 100,000 for White individuals. Despite these improvements, the disparity in CVD mortality between Black and White adults remained consistent, with a rate ratio of 1.31 (95% CI, 1.30-1.32) throughout the study period, indicating no significant change in the relative risk.

In the Atherosclerosis Risk in Communities Study (ARIC) research, Zhao et al. examined a cohort comprising 3,832 Black participants and 11,237 White participants [18]. Their findings indicated that Black individuals, particularly women, faced a significantly higher risk of sudden cardiac death (SCD) compared to their White counterparts. Notably, the lifetime cumulative incidence of SCD by the age of 85 was higher among Black men at 9.6%, compared to 6.6% for Black women, 6.5% for White men, and 2.3% for White women. The study further investigated contributing factors and demonstrated that, beyond traditional risk factors, socioeconomic elements such as income and education also play a role in outcomes.

During the COVID-19 pandemic in the United States, Wadhera et al. noted significant increases in heart disease and cerebrovascular disease mortality across Black, Hispanic, Asian, and White populations [19]. The Black community saw the most significant increases, with heart disease mortality rising from 1,503.8 to 1,783.7 per million and cerebrovascular mortality from 379.7 to 430.7 per million from 2019 to 2020. Hispanic populations experienced increases in heart disease deaths from 820.4 to 968.5 per million and in cerebrovascular deaths from 235.9 to 264.4 per million. For non-Hispanic Asian populations, heart disease deaths increased from 577.4 to 685.7 per million, and cerebrovascular deaths rose from 207.4 to 236.5 per million. Non-Hispanic White populations also reported increases, with heart disease deaths growing from 1,208.7 to 1,234.2 per million and cerebrovascular deaths from 258.2 to 268.7 per million. These findings underscore the heightened vulnerability and impact of the pandemic on the Black, Hispanic, and Asian populations in terms of heart-related and cerebrovascular mortalities.

Evaluating cardiovascular healthcare barriers in the context of race

Most Studied Socioeconomic Factors: Education, Income, Employment, and Environment

Approximately 80% of the global burden of CVD is accumulated in low- and middle-income countries [20]. Lower SES not only correlates with CVD but also increases cardiovascular risk similarly to traditional factors such as hypertension, diabetes, obesity, and smoking [5]. Four key SES indicators, namely, education, income level, employment status, and environmental conditions, have demonstrated associations with CVD risk [21-24]. In addition, a single marker of SES is unlikely to predict CVD risk due to the complex interplay among various SES factors [6].

Education correlates with CVD outcomes [25]. Research indicates that in low- and middle-income countries, individuals with lower educational levels experience higher rates of incidence and mortality due to CVD [26]. Liu et al. observed an inverse relationship between education level and CVD risk factors; notably, reduced educational attainment is associated with increased levels of hypertension, body weight, smoking prevalence, and ECG abnormalities [21]. Over the decade from 2011 to 2021, educational completion rates for adults aged 25 and older improved across all ethnic groups [27]. Specifically, high school completion rates increased from 92.4% to 95.1% among non-Hispanic White individuals, from 84.5% to 90.3% among Black individuals, from 88.6% to 92.9% among Asian individuals, and from 64.3% to 74.2% among Hispanic individuals. Despite these gains, Black and Hispanic populations continue to exhibit the lowest educational completion rates compared to other groups. Additionally, Williams et al. reported that among racial groups, Black and Hispanic populations typically have the lowest educational levels of all races [28].

A study by Minhas et al. involving 35,932 participants revealed that lower income independently correlates with a higher likelihood of diabetes, hypertension, coronary artery disease, congestive heart failure, and stroke [29]. Furthermore, research by Shin et al. on 479,359 participants identified a significant association between low income and increased cardiovascular mortality (HR: 1.31; 95% CI, 1.25-1.38), as well as cardiovascular events (HR: 1.07; 95% CI, 1.05-1.10) among hypertensive patients [30]. Additionally, an analysis of U.S. Census data from 2000 to 2014 by Akee et al. points to a persistent income disparity, with White and Asian populations generally at the higher end of the income spectrum, while Black, American Indian, and Hispanic populations are disproportionately represented at the lower end [31].

Meneton et al. conducted a study involving 5,852 subjects and found an increase in cardiovascular event risk (HR: 1.84, 95% CI, 1.15-2.83, p = 0.01) and all-cause mortality (HR: 2.79, 95% CI, 1.66-4.47, p = 0.0002) among unemployed individuals compared to employed individuals [23]. Dupre et al. reported similar results in their study of 13,451 individuals, finding a significantly higher risk of acute myocardial infarction among the unemployed compared to those who were employed (HR: 1.35, 95% CI, 1.10-1.66, p < 0.001) [32]. The U.S. Bureau of Labor Statistics reports that among racial groups, unemployment rates were highest among Black populations (8.6%), followed by American Indian and Alaska Native populations (8.2%), Hispanic populations (6.8%), Asian populations (5.0%), Native Hawaiian and Other Pacific Islander populations (6.9%), and White populations (4.7%) [33].

Neighborhood amenities, including sidewalks, access to healthy foods, safety, social support, and community cohesion, are essential in shaping the character of neighborhood environments [34]. These elements are key to promoting and maintaining a healthy lifestyle. Neighborhood socioeconomic characteristics, in addition to individual SES, have been associated with CVD risk factors, adverse events, and mortality [6]. Diez Roux's research on 13,009 participants showed that residents of disadvantaged neighborhoods experienced a higher incidence of coronary heart disease for White populations (HR: 3.1, 95% CI, 2.1-4.8) and for Black populations (HR: 2.5, 95% CI, 1.4-4.5) [24]. These results are particularly notable as they reveal a somewhat unusual trend where White populations in disadvantaged neighborhoods exhibit a higher incidence of coronary heart disease compared to Black populations. Furthermore, residing in impoverished neighborhoods may correlate with heightened exposure to air pollution. An expanding body of research suggests a connection between air pollution and endothelial dysfunction, potentially contributing to the development of atherosclerosis [35].

Other Socioeconomic Factors: Food and Housing Insecurity and Insurance

A strong correlation exists between food insecurity and the prevalence of CVD and its risk factors in adults [36]. Food insecurity often leads to a poor diet, causing metabolic derangements and insulin resistance, and frequently coincides with psychological distress that may drive individuals toward unhealthy coping mechanisms such as smoking [37, 38]. In addition, housing insecurity may result in psychological distress, sleep deprivation, and heightened exposure to smoke, all of which can elevate the risk of CVD and increase mortality [39]. Homeowners often experience higher levels of happiness than renters and individuals dealing with housing insecurity, potentially reducing stress, a significant risk factor for cardiovascular health [40]. In 2020, approximately 10.5% of U.S. households were affected by food insecurity, with significantly higher rates in Black and Hispanic households [41]. Additionally, insurance plays a critical role in overcoming barriers to cardiovascular healthcare; uninsured adults not only face challenges in accessing healthcare and receive lower quality care but also exhibit poorer health outcomes and lower control and treatment rates for hypertension and hypercholesterolemia compared to those who are insured [42, 43]. Lastly, Black and Hispanic populations have lower insurance coverage rates than White populations across all age groups, which makes them more susceptible to the adverse health events mentioned previously [44].

Mental Health

The connection between mental health and CVD is well-established and operates in both directions. Mental health disorders can arise as complications of heart disease, and existing mental health issues can increase the risk of developing CVD [45, 46]. Cardiovascular conditions like stroke, myocardial infarction, and heart failure are known to precipitate mental health challenges such as depression, anxiety, and PTSD [47]. These psychological issues can lead to harmful behaviors, including a sedentary lifestyle, smoking, and poor adherence to medical treatments, further increasing the likelihood of cardiovascular complications [48]. Moreover, effective management of mental health conditions such as depression and anxiety has been linked to reduced hospitalizations for CVD, highlighting the importance of comprehensive mental health care in cardiovascular risk reduction [49]. Additionally, there are pronounced racial disparities in the prevalence of mental health conditions, with depression being notably more prevalent among Black and Hispanic individuals [50]. This disparity is compounded by the fact that ethnic minorities are generally less likely to access mental health services compared to their Caucasian counterparts, partly due to cultural barriers and a lack of services tailored to their specific needs [51]. This underutilization of mental health care emphasizes the need for culturally competent approaches that recognize the unique cultural contexts of minority groups, thereby improving both mental and cardiovascular health outcomes.

Research demonstrates the profound influence of SES on CVD risk and outcomes, particularly among Black and Hispanic populations [52]. Decreasing SES disparities offers a path to reduce CVD morbidity and mortality in the population. Further research into how SES affects cardiovascular health and refining risk assessments are essential. By focusing on these areas, we could develop targeted interventions to address the specific needs of diverse populations. Such strategies should improve cardiovascular health and ensure care advances are equitably distributed. This would lead to a more just health system where everyone can have a healthier life, regardless of socioeconomic or racial background.

## Conclusions

The study underscores the link between lower socioeconomic status, including classic factors like education, income, employment, and living conditions, and increased cardiovascular risks. It further examines the roles of food and housing insecurity and insufficient insurance coverage in compounding these risks. The study also explores the influence of mental health conditions, such as depression and anxiety, on cardiovascular health. A critical analysis is provided on the prevalence of these factors across various racial and ethnic groups, emphasizing the significant disparities. This highlights the critical need for comprehensive interventions and targeted policy reforms that address both the biological and socioeconomic factors to effectively decrease the disparities in cardiovascular disease prevalence and outcomes, particularly among the most vulnerable populations.
